# A case report of neurosyphilis coexisting with a positive MOG antibody manifested as optic neuritis

**DOI:** 10.3389/fneur.2023.1258043

**Published:** 2023-09-29

**Authors:** Min Shi, Danqing Luo, Zhaoying Li, Man Li, Shuoguo Jin, Dongdong Yang, Jun Guo, Guo Chen

**Affiliations:** ^1^Department of Neurology, Hospital of Chengdu University of Traditional Chinese Medicine, Chengdu, China; ^2^Department of Neurology, Chongqing Hospital of Traditional Chinese Medicine, Chongqing, China; ^3^Department of Infectious Diseases, Hospital of Chengdu University of Traditional Chinese Medicine, Chengdu, China

**Keywords:** neurosyphilis, MOG, MOGAD, optic neuritis, *Treponema pallidum*

## Abstract

**Background:**

Neurosyphilis refers to an infection of the central nervous system by *Treponema pallidum*. The clinical manifestations of neurosyphilis are diverse, making it easy to miss or misdiagnose. Anti-myelin oligodendrocyte glycoprotein antibody-associated disease is a recently defined immune-mediated inflammatory demyelinating central nervous system disease. Few studies have reported the coexistence of the two diseases.

**Case presentation:**

This case report presents a 37 years-old male patient with neurosyphilis manifested as optic neuritis with a positive myelin oligodendrocyte glycoprotein (MOG) antibody. This patient received intravenous administration of 3.2 million units of procaine penicillin every 4 h for 2 weeks, followed by a two-week intramuscular injection of benzathine penicillin. Additionally, methylprednisolone sodium succinate was administered intravenously at 1,000 mg/day, gradually reduced to 500 mg/day and 240 mg/day every 3 days. Subsequently, prednisone tablets at a dosage of 60 mg/day were orally administered, with a gradual reduction of 5 mg/day every 3 days until reaching a dosage of 30 mg/day. The patient’s visual acuity was improved after 26 days of hospitalization. However, the visual field and color vision did not. At 3 months of follow-up, the symptoms remained unchanged despite the patient continued taking oral prednisone tablets at a dosage of 30 mg/day.

**Conclusion:**

Neurosyphilis could be a potential triggering factor for MOGAD. In patients with neurosyphilis, it is strongly recommended to perform testing for MOG antibody along with other brain disease antibodies.

## Introduction

Neurosyphilis is an infection of the central nervous system (CNS) by syphilis spirochete, and its clinical manifestations are diverse. Ocular symptoms are specific manifestations of neurosyphilis (1), which can occur at any stage of the disease and damage multiple eye structures (2, 3). Anti-myelin oligodendrocyte glycoprotein immunoglobulin G (IgG) antibody-associated disease (MOGAD) is an immune-mediated inflammatory demyelinating disease of the CNS. Optic neuritis is one of the main symptoms of MOGAD (4). Few studies have reported the coexistence of neurosyphilis and positive myelin oligodendrocyte glycoprotein (MOG) antibodies. It remains unknown whether the clinical manifestations, treatments, and prognosis of patients with neurosyphilis or positive MOG antibody alone differ from those of patients with both diseases.

In this case report, we presented a male patient with neurosyphilis manifested as optic neuritis and positive MOG antibody. He tested positive for both serum *Treponema pallidum*-specific antibody and MOG antibody. The visual acuity was successfully restored after 26 days treatment with penicillin and corticosteroids, while the visual field and color vision impairments were not ameliorated.

## Case presentation

A 37 years-old male patient was admitted to our hospital with blurred vision in both eyes for over a year and an aggravation of the symptoms for a month. He had experienced decreased visual acuity and color vision disorder in both eyes with unknown cause for over a year. The patient stated that he could not see the traffic light clearly when crossing the street. He was diagnosed with chorioretinopathy in both eyes at a local hospital but did not receive standard treatment. Since then, the patient has visited different hospitals successively and taken methylcobalamin supplements, but without significant response. This patient reported no history of chronic or genetic diseases. He visited sex workers twice before admission. One occurred 1 year ago, and the other occurred 2 months ago.

The examination results in other hospitals are as follows: (1) Fundus angiography (3 months after onset): the circulation time was roughly normal. Optic disc fluorescence was fair in both eyes. Dotted translucent fluorescence was observed in the retina of the right eye. Subretinal fluorescence was stained in the late circulation of both eyes. The diagnosis was chorioretinopathy in both eyes. (2) Visual evoked potential (VEP) (7 months after onset): the VEP was abnormal in both eyes. Conduction disorder of the visual pathway was detected, especially in the right eye. (3) Optical coherence tomography (7 months after onset): the mean thickness of RNFL: OD, 66 μm; OS, 70 μm. The symptoms at admission were blurred vision in both eyes with color vision disorder but no optical rotation, double vision, dizziness, headache, or other discomforts.

The results of physical examination at admission in our hospital are as follows: Body temperature, 36.8°C; heart rate, 84 beats/min; respiratory rate, 20 breath/min; blood pressure, 118/74 mmHg. The patient had no rash on the skin, mucous membranes of the whole body, or enlargement of superficial lymph nodes. He exhibited Argyll Robertson pupils: bilateral pupils significantly constricted with a diameter of approximately 2 mm; weak direct and consensual light reflex; normal near reflex. The visual acuity was determined by examining whether the patient could recognize hand movement in front of the right eye and count fingers 50 cm from the left eye. This patient also presented significant color vision changes.

The urine routine, stool routine, coagulation, liver and kidney function, electrolyte, and levels of C-reactive protein, blood lipid, blood glucose, and myocardial enzyme at admission were normal. This patient tested positive for serum *Treponema pallidum*-specific antibody, with a 1:32 (Trust test) titer on the third day of admission. The fluorescent treponemal antibody absorption (FTA-ABS) test showed that the FTA-ABS-IgG and FTA-ABS-IgM antibodies were positive. The result of the human immunodeficiency virus infection (HIV) test was negative. The results of the cerebrospinal fluid (CSF) test were as follows: (1) pressure: 190 mmH_2_O; (2) white blood cells WBC: 59.0 × 10^6^/L, (3) neutrophil percentage: 3%; (4) lymphocyte percentage: 30%; (5) monocyte percentage: 67%; (6) Cl levels: 121.8 mmol/L; (7) glucose levels: 3.24 mmol/L; (8) protein levels: 1142 mg/L; (9) CSF IgG concentration: 34.3 mg/dL (Supplementary Table S1). The antibody was tested by flow cytometry using the EUROIMMUN kit (Germany) and the CBA-IF method of the Neurological Research Laboratory of Medical Innsbruck. The patient tested positive for the MOG antibody IgG (1:200) in both serum and CSF samples on the fifth day of admission. The serum and CSF samples also tested negative for anti-flotillin-1/2 antibody IgG, anti-MBP antibody IgG, and anti-AQP4 antibody IgG. The CSF was reexamined 1 week after treatment: (1) pressure: 135 mmH_2_O; (2) WBC: 44 × 10^6^/L; (3) Cl levels: 128.1 mmol/L; (4) glucose levels: 4.0 mmol/L; (5) protein levels: 881 mg/L; (6) CSF IgG concentration: 25.5 mg/dL (Supplementary Table S1). The patient tested positive for CSF anti-*treponema pallidum* antibody IgG, with a titer of 1:26 (FTA-ABS test).

**Figure 1:**
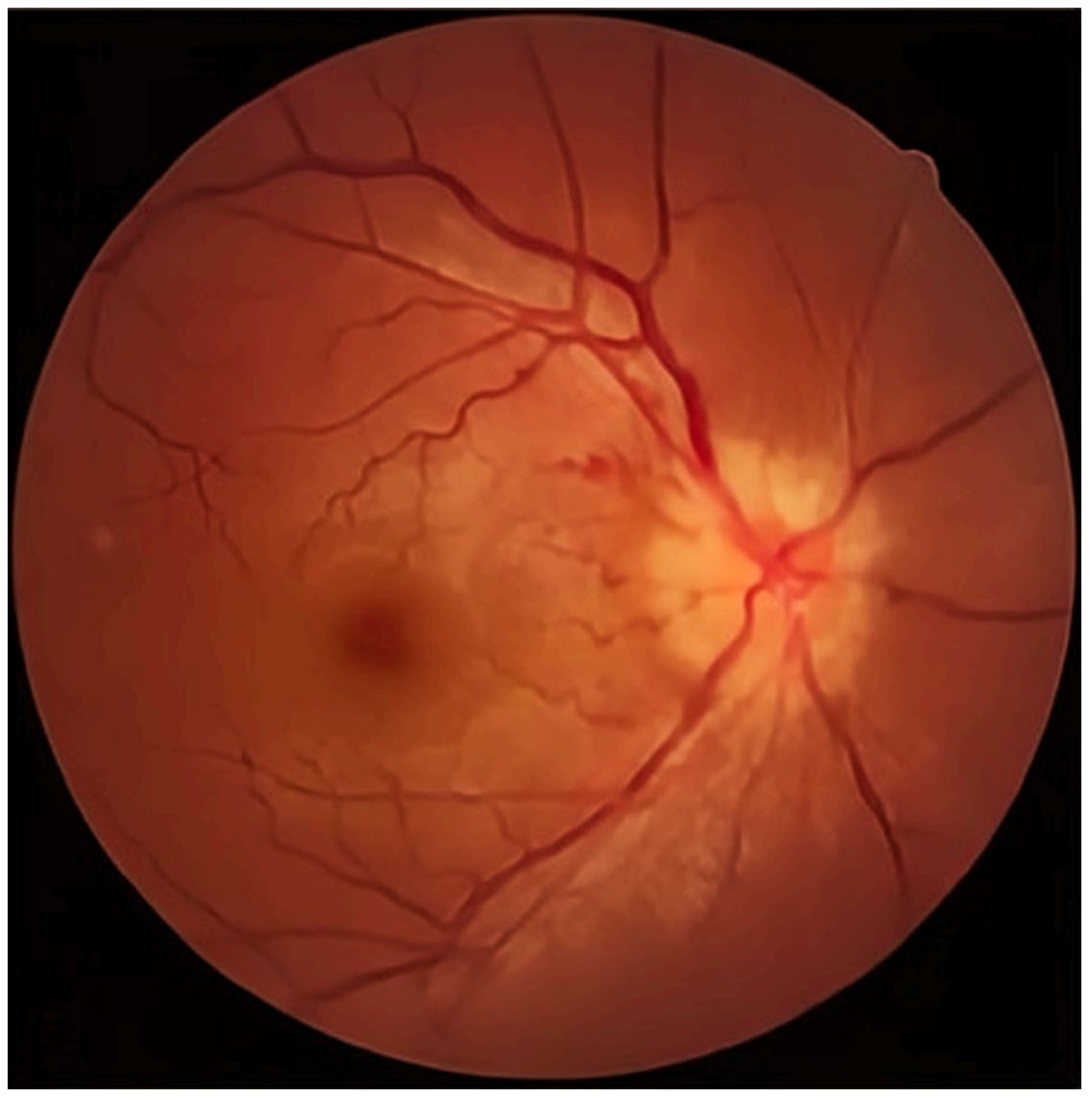
The fundus examination.

The visual acuity examination showed a visual acuity of 0.2 in the right eye and 0.15 in the left eye. The color vision test indicated red-green color weakness. The fundus examination revealed significant optic disc edema and blurred disc margins in the left eye (Figure 1).

**Figure 2:**
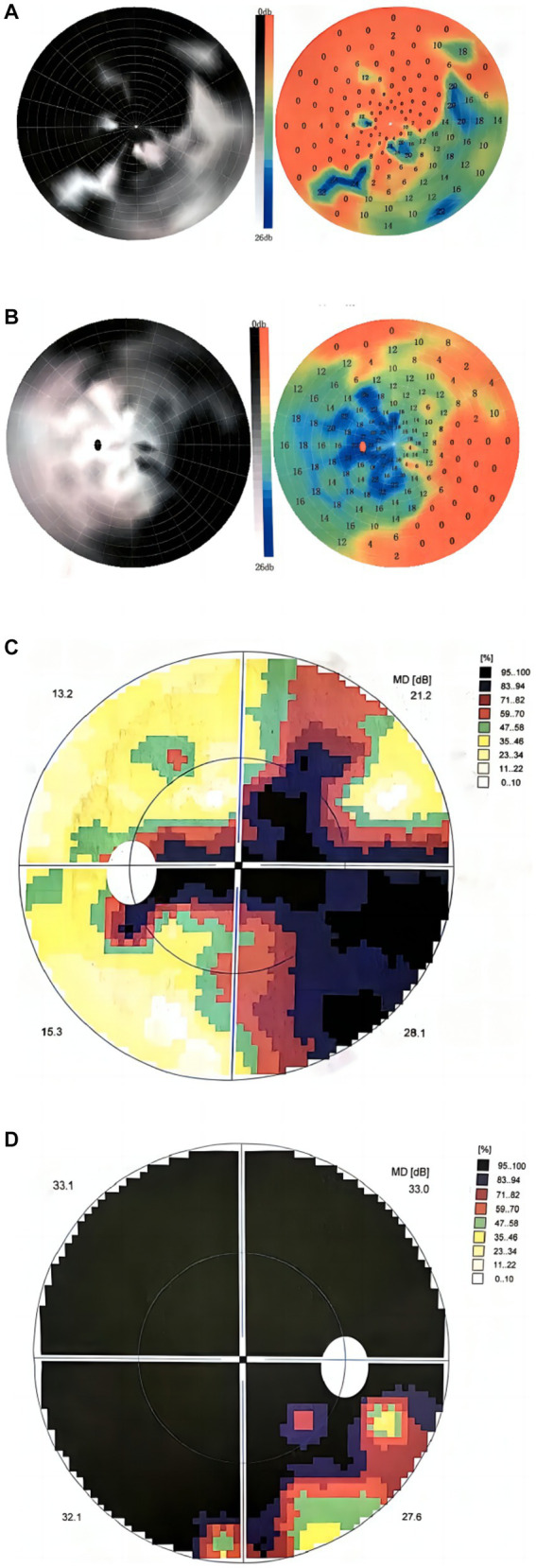
Visual field maps at 3 and 16 months after onset. **(A,B)** The visual field map 3 months after onset showed **(A)** an infratemporal insular visual field in the right eye, **(B)** central scotoma connected to the physiological scotoma in the left eye, and arcuate scotoma on the nasal side. **(C,D)** The visual field map at 16 months after onset showed **(D)** a smaller insular visual field in the inferotemporal region of the right eye, **(C)** central scotoma connected to the physiological scotoma in the left eye, and arcuate scotoma on the nasal side.

**Figure 3:**
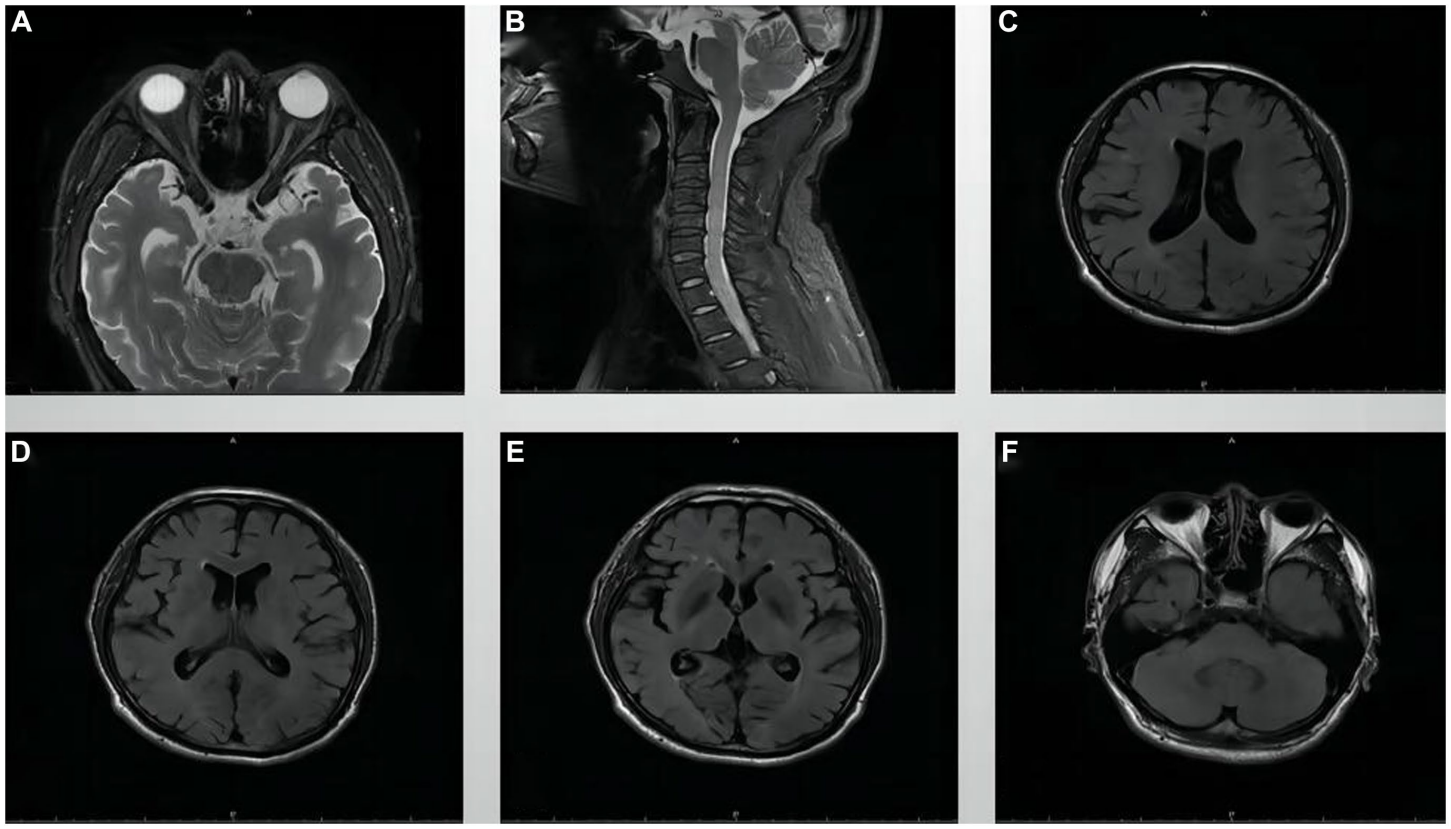
MRI images of the orbit, cervical spine, and brain. **(A)** Orbital MRI, **(B)** Cervical spine MRI, and **(C–F)** Brain MRI.

The visual field examination showed an insular visual field, referring to severe concentric narrowing with only a remaining visual field within a range of 5 to 10 degrees around the center, in the inferotemporal region of the right eye (Figures 2A,D), a central scotoma connected to the physiological scotoma in the left eye (Figures 2B,C), and an arcuate scotoma on the nasal side. The brain magnetic resonance imaging (MRI) and enhanced scan detected a few small microvascular lesions in the bilateral frontal lobe, parietal lobe, and right insular lobe. No abnormal signal was observed in the cerebellum or brainstem. No abnormal enhanced lesion was observed in the enhanced scan of the brain (Figures 3A,C–F). The cervical spine MRI and the enhanced scan revealed mild posterior median protrusion of the cervical intervertebral disc 3–4, 4–5, 5–6, and 6–7, cervical intervertebral disc degeneration, and mild hyperosteogeny of the cervical vertebral body 3–7 (Figure 3B). No abnormal enhanced lesion in the enhanced scan or abnormal signal in the cervical spinal cord was detected. No abnormality was observed in the orbital MRI.

**Figure 4:**
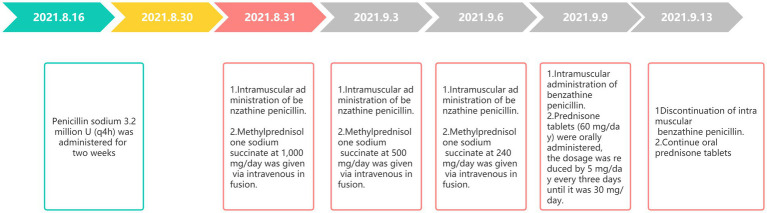
Treatment course.

The VEP showed poor bilateral P100 differentiation and delayed latency, suggesting abnormal bilateral visual pathway conduction. Based on the medical history and results of the auxiliary examination (Supplementary Table S2), the diagnosis of neurosyphilis combined with MOGAD was made. This patient received intravenous administration of 3.2 million units of procaine penicillin every 4 h for 2 weeks, followed by intramuscular injection of benzathine penicillin for an additional 2 weeks. The examination results showed serum *Treponema pallidum* (+) with a titer of 1:8 (TRUST test) and cerebrospinal fluid *Treponema pallidum* (+) with a titer of 1:5. Penicillin sodium at 3.2 million U (q4h) was administered for 2 weeks. Then, methylprednisolone sodium succinate at 1,000 mg/day was given via intravenous infusion. The dosage was successively reduced to 500 and 240 mg/day every 3 days. Next, prednisone tablets (60 mg/day) were orally administered, and the dosage was reduced by 5 mg/day every 3 days until it was 30 mg/day. When corticosteroid treatment started, penicillin sodium treatment was replaced by intramuscular administration of benzathine penicillin, which lasted for 2 weeks (Figure 4). The patient’s visual acuity (0.8 in the right eye, 0.6 in the left eye) were improved after 26 days of hospitalization. However, the visual field and color vision did not. Then, the patient and his family requested discharge and refused lumbar puncture. The CSF examination was not performed before discharge. The patient was advised to take prednisone tablets (30 mg/day) orally and continue the treatment after discharge. The symptoms were not significantly improved at 3 months of follow-up. This patient exhibited no meningeal irritation signs during the disease.

## Discussion and conclusions

Neurosyphilis can occur at any stage of syphilis infection with four common clinical subtypes: asymptomatic neurosyphilis, meningeal neurosyphilis, meningovascular syphilis, and paralytic dementia (5). Approximately 5% of untreated syphilis patients develop neurosyphilis (6), and the incidence of neurosyphilis of various manifestations is around 0.47–2.1 per 100,000 population (7, 8). Ocular syphilis is a subtype of neurosyphilis, accounting for 2–10% of all syphilis cases (9). Syphilis is a bacterial infection that can damage any structure of the eye (2), and approximately 20% of the cases with damage involve the optic nerve (10). Anti-myelin oligodendrocyte glycoprotein antibody-associated disease (MOGAD) is an inflammatory demyelinating disease of the CNS, with the most common manifestation being optic neuritis, followed by myelitis, acute disseminated encephalomyelitis (ADEM), and ADEM-like manifestations (11).

Previous studies have reported cases of CNS infectious diseases combined with immune-mediated diseases, such as neurosyphilis combined with AQP4 antibody-positive neuromyelitis optica spectrum disorders (12), neurosyphilis combined with anti-N-methyl-D-aspartate receptor encephalitis (13), herpes simplex encephalitis combined with autoimmune encephalitis (14), and SARS-CoV-2 combined with MOGAD (15, 16). These findings suggest that viral infection may be related to initiating and developing immune-mediated diseases. Whether producing virus-induced autoimmune antibodies may induce immune responses warrants further investigation.

In this case report, we presented a patient with both neurosyphilis and MOGAD. *Treponema pallidum* can invade the CNS via the blood or lymphatic system, inducing inflammatory reactions and disrupting the blood–brain barrier. However, the underlying mechanisms remain unclear. Considering the medical history, physical examination, and imaging of this patient, as well as his two visits to sex workers (one occurred 1 year ago and the other occurred 2 months ago before admission), we speculated that this patient might have developed chorioretinopathy caused by neurosyphilis, and the disease had a slow progression. He had blurred vision in both eyes for over a year and an aggravation of the symptoms for a month. The rapid onset was different from that of neurosyphilis-induced oculocutaneous syphilis. The symptoms, including acute loss of binocular vision, visual field defects, color vision changes, etc., suggested that it might be an acute phase of MOGAD-ON. Therefore, we considered that the etiology of optic neuritis as a combination of neurosyphilis and MOGAD, and neurosyphilis could be a potential triggering factor for MOGAD. The combination treatment of corticosteroids and penicillin successfully restored the patient’s visual acuity but did not ameliorate the visual field and color vision impairments. Considering that high-dose corticosteroid is not recommended in treating neurosyphilis (17, 18), we initially treated this patient with standard antisyphilitic treatment, followed by high-dose corticosteroid. Further serum and cerebrospinal fluid antibody tests were not performed because this patient requested discharge. While the patient’s visual acuity improved, there were no significant changes in visual field and color vision. Subsequent telephone follow-ups indicated a relatively stable condition.

A recent report by Gudenkauf et al. (19) described a case of meningoencephalomyelitis associated with MOG-IgG seropositivity in a patient with syphilis. While both their case and ours involved syphilis infection and MOG-IgG seropositivity, our case involves the coexistence of two distinct etiologies and had a more precise diagnosis, offering a more clinically valuable and standardized therapeutic regimen. Firstly, in our case, a series of optic neuritis episodes occurred, starting with neurosyphilis-related optic neuritis and followed by MOGAD-related optic neuritis. The unique disease progression appeared to be closely linked to the patient’s visits to sex workers. In contrast, the case reported by Gudenkauf et al. only presented symptoms related to MOGAD-associated meningoencephalomyelitis. Moreover, this patient experienced additional symptoms, including fever, nausea, vomiting, diarrhea, body aches, and headache between the second and third doses of penicillin therapy, raising the possibility of influenza infection. Therefore, it cannot be asserted that syphilis infection mediated the development of MOGAD-associated meningoencephalomyelitis. Secondly, the diagnosis in our case was based on positive anti-*treponema pallidum* antibody IgG in both blood and CSF samples. Additionally, clinical symptoms and signs, as well as funduscopic examination findings supported the diagnosis. However, in their case, neither syphilis nor MOG antibody in CSF were tested. Thirdly, the treatment regimen in our case was carefully considered, taking into account potential conflicts in treating optic neuritis due to syphilis and MOGAD-associated optic neuritis. Therefore, we opted for a comprehensive approach, beginning with antisyphilitic therapy, followed by corticosteroid treatment. The treatment for syphilis was consistently maintained throughout the process. The outcomes of this treatment regimen were highly favorable, resulting in a significant improvement in the visual acuity of this patient. This treatment regimen could serve as a valuable guideline for patients with similar presentations.

In conclusion, neurosyphilis may be a triggering factor for MOGAD. We recommend testing the MOG antibody and other brain disease antibodies, such as AQP4 and NMDAR antibodies, in patients with neurosyphilis. Treatment with penicillin and corticosteroids is recommended, but attention should be given to the sequences of their administration.

## Patient perspective

This study was approved by the Ethical Review Committee of the Hospital of Chengdu University of Traditional Chinese Medicine (Chengdu, China) (Approval no. 2019KL-061). All procedures were per the Journal of International Medical Research, the ethical standards of the institutional and/or national research committee, and the 1964 Helsinki Declaration and its later amendments or comparable ethical standards. Written informed consent to participate in this study was provided by the patient. Written informed consent was also obtained for publishing any potentially identifiable images or data included in this article.

## Data availability statement

The original contributions presented in the study are included in the article/Supplementary material, further inquiries can be directed to the corresponding authors.

## Ethics statement

The studies involving human participants were reviewed and approved by the Ethical Review Committee of the Hospital of Chengdu University of Traditional Chinese Medicine (Chengdu, China). The patients/participants provided their written informed consent to participate in this study. Written informed consent was obtained from the individual(s) for the publication of any potentially identifiable images or data included in this article.

## Author contributions

MS: Conceptualization, Data curation, Writing – original draft. DL: Data curation, Writing – original draft. ZL: Data curation, Writing – original draft. ML: Data curation, Writing – review & editing. SJ: Investigation, Writing – review & editing. DY: Investigation, Writing – review & editing. JG: Conceptualization, Supervision, Writing – review & editing. GC: Conceptualization, Supervision, Writing – review & editing.

## Abbreviations

CNS: central nervous system
IgG: immunoglobulin G
MOG: myelin oligodendrocyte glycoprotein
MOGAD: myelin oligodendrocyte glycoprotein antibody-associated disease
VEP: visual evoked potential
FTA: fluorescent treponemal antibody
HIV: human immunodeficiency virus
CSF: cerebrospinal fluid
MRI: magnetic resonance imaging
ADEM: acute disseminated encephalomyelitis
