# Supplementary material for: A case report of neurosyphilis coexisting with a positive MOG antibody manifested as optic neuritis
Source: Front Neurol. 2023 Sep 29;14:1258043. doi: 10.3389/fneur.2023.1258043 (PMC10583717; doi:10.3389/fneur.2023.1258043)
Supplement: Supplementary file 1 [file Table_1.DOCX]

**Supplementary Table 1. The results of CSF test before and after treatment**

| **Items** | **At admission** | **After one-week treatment with corticosteroids** |
| --- | --- | --- |
| **Pressure (mmH_2_O)** | 190 | 135 |
| **WBC (× 10^6^/L)** | 59.0 | 44 |
| **Cl (mmol/L)** | 121.8 | 128.1 |
| **Glucose (mmol/L)** | 3.24 | 4.0 |
| **Protein (mg/L)** | 1142 | 881 |
| **IgG (mg/dL)** | 34.3 | 25.5 |

**Supplementary Table 2.** **The abnormal results of the auxiliary examination**

| **Items** | **Results** |
| --- | --- |
| **Mean corpuscular volume** | 102.6 fL |
| **Mean corpuscular hemoglobin** | 35.1 pg |
| **Red blood cell distribution width** | 44.2 fL |
| **Urine pH** | 7.0 |
| **Stool** | (-) |
| **Plasma fibrinogen** | 1.97 |
| **Aspartate aminotransferase** | 14 U/L |
| **γ-glutamyltransferase** | 9 U/L |
| **High-density lipoprotein cholesterol** | 0.88 mmol/L |
| **Apolipoprotein A1** | 0.87 g/L |
| **Cystatin C** | 1.14 mg/L |
| **Electrolytes** | (-) |
| **Blood glucose** | (-) |
| **Myocardial enzyme** | (-) |
| ***Treponema pallidum*-specific antibody** | (+) Positive (1:32) |
| **FTA-ABS-IgG** | (+) |
| **FTA-ABS-IgM** | (+) |
| **CSF anti-*treponema pallidum* antibody IgG** | (+) Positive (1:32) |
| **Serum anti-AQP4 antibody IgG** | (-) |
| **Serum anti-MOG antibody IgG** | Positive (1:200) |
| **CSF anti-MOG antibody IgG**  **Electrocardiogram** | Positive (1:200)  Sinus arrhythmia, normal ECG |
| **Chest computed tomography** | (-) |
| **Brain MRI scan + enhanced scan** | A few small microvascular lesions in the bilateral frontal lobe, parietal lobe, and right insular lobe |
| **Cervical spine MRI scan + enhanced scan** | Mild posterior median protrusion of cervical intervertebral disc 3-7, cervical intervertebral disc degeneration, and mild hyperosteogeny of cervical vertebral body 3-7 |
| **Orbital MRI** | (-) |
| **VEP** | Poor bilateral P100 differentiation and delayed latency |
